# Alveolar Echinococcosis—A Challenging Task for the Hepatobiliary Surgeon

**DOI:** 10.3390/pathogens11010040

**Published:** 2021-12-31

**Authors:** Jens Strohaeker, Mihaly Sulyok, Alfred Koenigsrainer, Silvio Nadalin

**Affiliations:** 1 Department of General, Visceral and Transplantation Surgery, University Hospital of Tuebingen, 72076 Tuebingen, Germany; Alfred.Koenigsrainer@med.uni-tuebingen.de (A.K.); Silvio.Nadalin@med.uni-tuebingen.de (S.N.); 2 Department of Pathology and Neuropathology, University Hospital Tuebingen, 72076 Tuebingen, Germany; Mihaly.Sulyok@med.uni-tuebingen.de

**Keywords:** alveolar echinococcosis, echinococcus multilocularis, hepatectomy, major liver resection

## Abstract

(1) Background: Alveolar echinococcosis (AE) is an ultimately fatal disease, whose only curative treatment is surgery. Due to its late presentation extended liver resections are often necessary. The true benefit of extensive surgery has yet to be established; (2) Methods: We present a single center experience of 33 cases of Echinococcus multilocularis that have been treated at a high-volume hepatobiliary surgery center between 2004 and 2021. (3) Results: Of the 33 patients 24 patients underwent major liver resection (73%). In addition to the liver resection patients frequently underwent complex extrahepatic procedures such as lymphadenectomy (n = 21, 61%), vascular resections and reconstructions (n = 9, 27%) or resections and reconstruction of the extrahepatic bile duct (n = 11, 33%). Seven patients suffered from ≥ grade III complications (21%). Complete resection was achieved in 17 patients. Fourteen patients had R1 resections and two had macroscopic parasitic remnant (R2). Progressive disease was reported in three patients (The two R2 patients and one R1 resected patient). At a median follow-up of 54 months no mortality has occurred in our cohort; (4) Conclusions: Liver resection remains the gold standard for AE. Even in extensive disease the combination of complex resection and perioperative benzimidazoles can achieve favorable long-term outcomes.

## 1. Introduction

Alveolar echinococcosis (AE) is a rare parasitic infection caused by *E. multilocularis* that mainly affects central and eastern Europe, parts of Turkey, and Western Asia [1]. The most commonly infected organ is the liver from where the parasites spreads via the hematogenous route or continuous infiltration. Left untreated it is fatal in up to 90% of patients over 10 years [2,3,4]. Historically the zoonosis has been treated surgically until the benzimidazoles (BMZ) were introduced as parasitostatic treatment alternatives in the 1970s and 1980s [5]. While the only cure of the disease is complete resection (followed by adjuvant medical therapy) the beneficial outcomes of “palliative” medical treatment has become the main alternative to surgery predominantly in patients of advanced age and disease. The introduction of BMZ has lifted the 10-year survival rate from around 10 percent to over 80 percent for upfront inoperable cases [3,6,7]. More than ever is the treatment course set by infectious disease specialists and parasitologists and moved out of the surgeon’s focus [8]. Even though the incidence in Germany remains low (0.63/100,000 inhabitants) the repopulation of foxes to both rural and urban areas in the last decades has contributed to increasing case numbers [9,10,11]. 

In spite of the increasing incidence, AE still remains a neglected disease. Patients are frequently misdiagnosed as benign or malignant tumors or falsely identified as cystic echinococcosis (CE) and subsequently treated suboptimally [12]. Therefore, several recent studies have tried to put the disease back into the clinician’s focus [13,14,15]. 

The most potent antihelmintic drug for AE is the BMZ Albendazole (ABZ). Even though it was approved in 1982 for intestinal parasitosis, it was never licensed as a treatment for AE and is therefore considered off-label treatment [16]. While no new drugs for AE have been developed in the last four decades, liver resections have become safer since the 1980s with better resection techniques and surgical tools, increased understanding of liver regeneration as well as remnant liver functional capacity and consequently lower risk of post-hepatectomy liver failure (PHLF) [17]. The current WHO-Guidelines do recommend abstaining from surgery for most patients with locally advanced PNM stages especially with infiltration of neighboring structures, locoregional lymph node positivity (N+) or metastatic disease (M+) [18,19]. Therefore, there is an ongoing discussion on how aggressive surgical procedures should really be.

While BMZ are generally well tolerated drugs, up to 10% of patients develop acute liver toxicity during the course of treatment [7,20]. Patients who have undergone long-lasting BMZ treatment for AE may present with liver fibrosis or even cirrhosis, which can make hepatectomy a high-risk procedure for these patients. Fibrosing of the liver is likely caused by the combination of chronic parasitic inflammation and cholestasis. Whether BMZ toxicity (and subclinical liver enzyme elevations) aggravate this process is yet to be studied. Patients suffering from (repeated) BMZ toxicity and/or disease progression under therapy are frequently referred for the evaluation of salvage resection. 

From the surgeon’s perspective, there are several unsolved problems. *AE* often presents as an infiltrating tumorous mass that affects hilar vascular structures, bile duct and locoregional lymph nodes. To date the general recommendation is to abstain from vascular resections and lymphadenectomy both due to the increased risk of complications and the unclear role of lymphatic spread in parasitic recurrence [21,22]. Additionally, for major liver resections in AE patients the recommended safety margins of 20 mm are hard to achieve due to perivascular growth and the increased risk of posthepatectomy liver failure (PHLF) due to insufficient functional liver remnant [18,23,24]. The risk of PHLF increases when major liver resections are performed [25], remnant liver fibrosis or cirrhosis is present and if vascular exclusion is performed throughout the procedure [26]. 

The Department of General, Visceral and Transplantation Surgery of the University Hospital of Tuebingen is a tertiary hepatobiliary center with expertise in complex major liver resection for biliary tract malignancies and has served as a referral center for extended liver resections in often declared-unresectable AE manifestations as well as patients that were intolerant to medical treatment [27,28]. 

The main aim of this study is to present our results with liver resection in a German endemic area of AE. 

## 2. Results

From 2004 to July 2021 75 patients with AE were treated at our institution. Of these patients, 33 underwent liver resection 24 of them being major liver resections. The male to female ratio was 16 to 17. The mean age at resection was 52 years (Standard Deviation (SD) ± 14 years; median 54 years, range 19–82) (Table 1).

### 2.1. Diagnosis

None of the 33 patients presented in this cohort underwent core liver biopsy prior to exploration. The presumed diagnosis was AE in 64% (n = 21), CE in 18% (n = 6), intrahepatic cholangiocarcinoma (iCCA) in 9% (n = 3) and hepatic metastasis of colorectal carcinoma (CRC) in 9% (n = 3). Twenty-two patients (66%) were treated with BMZ (in our cohort we solely prescribed Albendazole) preoperatively for a median duration of 1 month (SD ± 26 months; range 0–146). In the three patients that were presumed to suffer from iCCA intraoperative frozen section ruled out iCCA and a diagnosis of AE was made. All three patients were resected according to the oncologic principles followed for cholangiocarcinoma. Similarly, the three patients that suffered from colorectal carcinoma were resected according to oncologic principles. In these three patients the diagnosis of AE was made postoperatively on histology.

### 2.2. Pretreatment

Twenty-two patients underwent preoperative BMZ treatment. Excluding two patients (One with less than four weeks of treatment and one with unknown treatment duration) twenty patients were pretreated with BMZ for at least one month (10 patients had cycled treatment whereas 10 were treated continuously). The overall median preoperative treatment duration for all patients was one month; for the 20 patients that were treated more than one month the median was 3 months. The longest preoperative treatment was 146 months.

Ten patients were pretreated for 1–3 months in a neoadjuvant intention whereas 10 were treated for >6 months in an attempt to treat the patients definitively with benzimidazoles. These 10 were ultimately referred to our department when the following problems arose: BMZ- toxicity (n = 5), disease progression (n = 2), other complications (n = 2: one cystocolonic fistula, one hepatic outflow obstruction). One patient suffered from adjustment disorder and was unable to move on with surgery for 6 months. 

### 2.3. Extent of Liver Resection

Of the 33 patients, 9 underwent minor liver resection (27%) while 24 underwent major liver resection (73%). Most resections were right hepatectomies and right trisectionectomies.

During these 33 procedures 21 patients underwent lymph node resections including 15 systematic radical lymphadenectomy (mainly hilar). 

Nine patients had vascular resection and reconstruction. Four portal veins appeared to be infiltrated by the parasite, one had additional portal vein thrombosis adjacent to the periportal parasitic lesions. Three of these were partially resected and underwent end-to-end reconstruction. The fourth underwent thrombectomy through the transected ostium of the resected liver part. In five cases a reconstruction of the Inferior Vena Cava (IVC) was necessary: two by primary suture closure, one with horizontal cavoplasty of the anterior surface of the IVC, one with a bovine pericardium patch repair and one with IVC replacement by means of a 28 mm GoreTex®-Prosthesis. 

Eleven patients (33%) underwent resection of the extrahepatic bile duct. Ten patients underwent a single hepatico-jejunostomy either for one duct or multiple ducts with ductuplasty. One patient had two separate hepatico-jejunostomies. All additional procedures are displayed in Table 2. 

**Figure 1:**
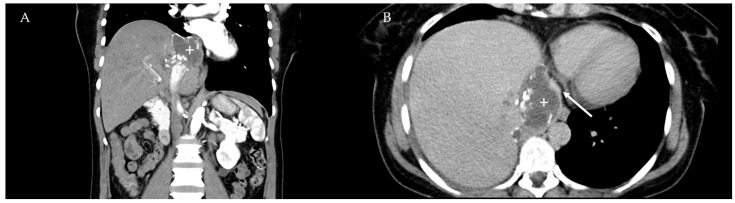
Computer tomography images of a patient with a locally advanced AE lesion (plus) in segment VIII of the liver (Image **A**, coronal view). Image **B** shows the close proximity to the inferior vena cava (arrow) that is nearly occluded by the parasite.

**Figure 2:**
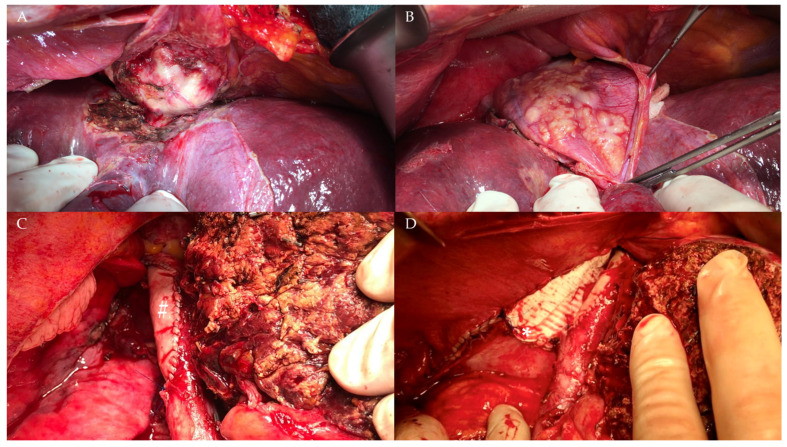
Intraoperative extent of the AE mass depicted in Figure 1. (**A**) parasitic infiltration of the wall of the inferior vena cava (IVC). (**B**) transdiaphragmatic infiltration of the parasite. (**C**) IVC reconstruction using a bovine pericardium patch (pound) placed under total vascular exclusion of the liver after right sided hepatectomy. (**D**) reconstruction of the diaphragm with a GoreTex® (W.L.Gore and Associates, Newark, DE, USA) patch (asterisk).

The median operative time was 283 minutes (SD ± 116 min; range 50–530). Overall, six patients underwent intraoperative blood transfusions and five had postoperative transfusions. The median number of packed red blood cells was 2 (SD ± 2.5, range 1–8). For a challenging surgical case see Figure 1 and Figure 2. 

### 2.4. Histopathological Results

We reexamined all histopathological specimens with an infectious disease pathologist. Overall R0 rate was 17/33 (52%) with overall R1 rate 14/33 (42%) and R2 resections in 2/33 (6%). R0 rate on the liver surface resection margin was 23/33 (69%). Median hepatic resection margin was 1 mm (SD ± 5.4 mm; range 0–20). In 21 procedures lymph nodes were resected. Ten of the 33 patients were nodal positive (30%). Twenty-three out of 141 lymph nodes were microscopically infested by the parasite on hematoxylin-eosin (HE) stain (16%). We did not perform immunohistochemistry and we did not consider a lymph node positive unless there was visible parasite or lamellar membranes on the HE stain. For the 15 patients who underwent systematic lymphadenectomy the median number of resected lymph nodes was eight.

### 2.5. Postoperative Course

No perioperative mortality was observed.

Of the 33 patients, 17 suffered from complications. Seven complications were ≥ IIIa according to Dindo/Clavien with five patients requiring reoperation (three bile leaks, one portal vein thrombosis and one bleeding). Complications were more common in major resections than minor resections (16 vs. 1; Χ^2^ *p* = 0.004).

The median length of postoperative stay was eleven days (SD ± 7 days; range 3–34).

Twenty-nine patients underwent adjuvant treatment with BMZ. Of the ones that completed their course, 12 had 24 months of BMZ two patients took BMZ for 18 months three for 3 months, two patients for 1 month. One patient took BMZ for 36 months, whereas two patients are on indefinite BMZ.

Of the 33 patients, 30 are still followed by our center. Three patients were lost to follow up. The last known status of these patients was alive and parasite free. All 30 patients are alive to date at a median length of follow up of 54 months (range 0–203). Overall, the intrahepatic recurrence rate of the disease was 1/33 (3%) whereas two patients underwent R2 resection. All three of these patients are alive and on indefinite BMZ therapy. 

### 2.6. Intraoperative “Surprises”

Cases of AE are full of surprises in different ways. In six patients we operated under the assumption of primary or secondary liver malignancy. Three patients underwent liver resection for suspected colorectal liver metastasis (CRLM). These three patients did not undergo liver hilum lymphadenectomy, for it is not standard of care for CRLM. Three other patients underwent liver resection with the suspicion of iCCA. In these patients AE was diagnosed intraoperatively through frozen sections. All three had lymph node dissection done even before the diagnosis was made and liver resection was commenced.

Aside from operating under false assumptions, one of the main challenges AE presents with, is how discrepant imaging can be to the intraoperative findings. Frequently imaging underestimates the true (hilar) extension of the disease. Comparing what was planned prior to resection to what was ultimately, done three patients (9%) had more extensive liver parenchyma resection than was expected preoperatively, while ten patients (30%) had more extensive extrahepatic vascular and bile duct reconstructions.

This is accompanied by marked inflammation especially in the liver hilum which makes dissection of liver vasculature demanding and dangerous. In one patient lymphadenectomy led to transection of an accessory right liver artery, which was successfully reconstructed. 

## 3. Discussion

Alveolar echinococcosis is a slowly advancing, but ultimately fatal disease if untreated [2,4]. While surgery is the only curative treatment option, the disease can be halted for decades with BMZ treatment [6,7]. Given that there are no national screening programs in central Europe (compared to few regions in Asia (e.g., central China [29] and Japan’s prefecture Hokkaido [30]), the disease is frequently discovered at an advanced stage [30]. Only 20–40% are considered to be resectable at the time of diagnosis [7,30,31]. Complete resection followed by adjuvant therapy with BMZ remains the gold standard for all resectable cases of non-metastatic alveolar echinococcosis. After a complete resection the adjuvant therapy should last for at least 24 months, if well tolerated, with a recommended follow-up of 10 years [18,32]. Whether this time can be shortened based on resection margins or should be prolonged based on hilar affection remains unclear.

Even in endemic areas AE lesions are frequently misinterpreted as hepatic malignancies, not to mention the non-endemic areas [12,15]. Stojkovic et al. recently presented data of the cases that were presented to their Echinococcus consultation service at the University of Heidelberg. In their study they reported on 80 patients they were consulted for. In 26 of these patients treatment decisions were made based on false assumptions [12]. Looking at our own data, we had similar issues. Of the 33 patients only 64% were correctly identified as AE preoperatively, 18% were mistaken for CE (mainly due to Serology or large “pseudocystic” lesions). The patients who underwent exploration for hepatic malignancy were run by the local tumor board, but not necessarily by radiologists dedicated to hepatic parasitoses. 

AE can only be diagnosed, taking into account the patients’ occupation, behavior, travel history and origin, daytime activities, serologic and imaging results, and, if needed, histology (Nowadays specific immunohistochemical antibodies exist for both species [22]). Due to the overall rarity of both AE and CE no interdisciplinary board discussing the treatment of these lesions has been established yet at our center, but may exist elsewhere. When in doubt we consider it no shame to refer the patient to a national referral service (for Germany these are either the University of Heidelberg for suspicion of CE or the University of Ulm for suspicion of AE). 

Inoperable patients should be treated indefinitely if BMZ are tolerated well. Reuter et al. have shown in 2004 that out of 15 patients with Positron Emission Tomography-negative lesions, who were discontinued, eight reactivated within 18 months [33]. Thus, treatment discontinuation with inactive lesions is possible but should not lead to cessation of follow-up.

Cyclic dosing is considered inferior to continuous BMZ intake. While continuous intake, has never been officially approved by the Food and Drug Administration (FDA) or European Medicines Agency (EMA) it is now considered the gold standard of medical treatment by all experts [34]. Reuter et al. presented their own experience with continuous and cyclic dosing and found no differences in toxicity [35] for which the drug was originally recommended to be interrupted every 28 days. 

The role of BMZ in a neoadjuvant setting has yet to be established. The current guidelines do not recommend neoadjuvant treatment, although there appears to be little harm to it. Even though both Albendazole and Mebendazole are considered parasitostatic [2], there are a few reports on successful downstaging of parasitic lesions in primarily unresectable cases that became radically resectable under therapy [14,36]. Additionally, patients can be evaluated for proper absorption of the drug and target doses, while also being screened for potential toxicity. Knowing about drug intolerance may also determine the targeted safety margins. When patients are stable under benzimidazole therapy and at low risk for complications, surgery may be postponed safely to a later point in time. A recent study by Schmidberger et al. compared the quality of life between patients that underwent curative resection compared to conservative medical treatment and came to the conclusion that medical treatment is not inferior to surgery with regards to mental and physical quality of life [37]. While we agree that liver resections are procedures with increased morbidity, frequently large incisions and potentially short and long-term consequences for the patients we still believe that medical treatment only should be reserved for people at an advanced age, when BMZ therapy offers the same “life expectancy” as surgery and to patients where incisional morbity or liver tissue loss outweighs the benefits of curative resections (e.g., small central or posterior right sided lesions). Additionally, more widespread use of laparoscopic and robotic liver resection may offer less invasive treatment options for smaller lesions in the future. 

Buttenschoen et al. presented some of the earliest modern surgical data when they compared their operative long-term results of the last century to the period following the year 2000. Diagnosing AE earlier in the recent years lead to more successful surgical procedures and R0-resections due to less advanced surgical stages in their cohort of 36 patients [38]. Today, we present a similar sized-cohort of patients that underwent liver resection for AE at a tertiary hepatobiliary center. Patient selection and modern resection and reconstructive surgical technique has drastically reduced gross incomplete (R2) resections; however, the more aggressive approach is accompanied by an increase of marginal (R1) resections. In order to pay tribute to the invasive behavior of the parasite, the width of resection margins on the liver surface have been a topic of discussion ever since. Historically, a minimum safety margin of 20 mm has been recommended by the WHO-Guidelines [18,32]. With the introduction of adjuvant benzimidazoles the minimum resection margins have been discussed repeatedly and margins < 20 mm are considered to be safe in patients tolerating adjuvant BMZ [23,24]. Hillenbrand et al. recently published the recurrence rates from their registry and found only a single recurrence in a patient with a resection margin > 1 mm, three years after surgery. While they reported on a total of 15 recurrences in 92 patients, they unfortunately did not report dosing and duration of adjuvant therapy duration for these patients who developed recurrence. While we strongly believe that patients intolerant to BMZ can be cured by larger safety margins the minimum remains unknown. Frequently the surgeon’s hands are tied by the adjacency to vascular structures. 

Preservation of liver tissue is crucial for the patient. For metastatic liver disease the focus has shifted from large resections to multiple atypical resections with small resection margins. When we find multiple superficial lesions, even in BMZ intolerant patients, we perform 1mm margins leaving anatomical resections for the time of recurrence.

Hilar marginal resections, to our knowledge, have never been addressed separately. They appear to be less of a problem than positive resection margins on the liver when it comes to parasitic recurrence as long as there is no gross disease remnant. 

However, while the role of lymphatic spread remains unclear there are a few things to consider when patients are referred for major hepatectomy. Chronic inflammation leads to lymphadenopathy as does lymph node infestation [22]. Hilar parasitic lesions may also appear to be lymphatic tissue when in fact it is solely parasitic. The marked lymphadenopathy in these patients needs to be dissected in order to reach vascular control at the liver hilum thus the surgeon should always be well trained and well prepared to perform systematic lymphadenectomy in AE cases. 

Studies performed by the department of pathology in Ulm, Germany, have shown that immunohistochemistry is able to detect parasitic particles in most of the enlarged lymph nodes even if no gross parasitic structure is visible, although the role of these so-called “SPEMS” remains unclear [22]. 

Systematic lymphadenectomy is one of the crucial skills for a hepatobiliary surgeon. Hilar lymphadenectomy in the hands of an unexperienced surgeon can cause tremendous damage to the patient such as bile duct injuries, hepatic artery dissection or severe bleeding. Since the first description of parasitic infiltration of a lymph node the role of hilar or interaortocaval lymph node dissection has remained unclear given the likely low risk of recurrence from lymphatic origin. Kawamura et al. reported only ~3% of lymph node metastasis in their cohort of >150 patients. Similar data has been published by Hillenbrand et al. from Ulm. They found lymphonodal parasite on HE-Stains in 7 of 43 patients that had lymph nodes removed in their cohort of 109 patients [21,24]. Given our more aggressive approach to partial and/or systematic lymph node dissection, we present the highest percentage of lymph node infestation published so far. 

Twenty-three of the 141 lymph nodes we resected during 21 procedures were infiltrated by the parasite. These 23 lymph node metastases were from 10 patients which resembles 30% of our cohort. Excluding the stages 1 and 2 (n = 9) almost half the patients had lymph node metastasis (45% 10/22). 

Since parasitic recurrence from lymph nodes has not been described yet the surgeon must take care not to put the patient at an unnecessary risk and should abstain from lymphadenectomy. 

Similar to the inflammatory lymphadenopathy, marked inflammation is present in perivascular tissue. While vascular invasion is a contraindication for resection to most surgeons, we rarely saw true vascular invasion on our histologic specimen. Dissecting these vessels from parasite and lymph nodes compares to the complexity of perihilar cholangiocarcinoma or repeated bile duct inflammation in cholangitis. Thus, sharp dissection of parasitic lesions from hilar vessels, liver veins or IVC can lead to bleeding and the surgeon must be trained to resect and reconstruct those in appropriate manner. 

We strongly believe that AE cases should be discussed similarly to oncologic cases by an interdisciplinary board and be referred to a center of expertise in hepatobiliary surgery to improve patient outcome. Unfortunately, this is not mandatory in Germany to date.

While liver resections can be safely performed in selected octogenarians [17,39] we do think that the excellent capability of BMZs to halt disease progression should make medical treatment the go-to standard for poor surgical candidates and most patients over the age of 70 who are not at risk of local complications. We recently performed a right hepatectomy with partial IVC resection in an 80-year-old female under total vascular exclusion who was intolerant to BMZ and showed progression threatening to cause hepatic outflow problems. While she had an uneventful postoperative course, we are convinced that surgeons should stay humble performing challenging resections in the elderly in order to avoid unnecessary harm.

While there is no true consensus on neoadjuvant treatment, there is consensus for adjuvant medical therapy for 24 months with continuous BMZ, preferably ABZ [7,35]. Whether this may be shortened based on resection margins or prolonged for R1 resections is unclear.

Defining resectability can be tedious work. Studies have shown that for CRLM there is a wide discrepancy between what is considered resectable by medical and surgical departments but also between hepatobiliary surgeons [40,41]. Additionally, constant reevaluation under palliative treatment has been shown to be beneficial for patients [42]. This appears to not only be a profound problem for CRLM but likely also for AE treatment. In patients who are unable to undergo curative resection, palliative (incomplete) resections were shown to successfully alleviate symptoms [43,44]. Recently Qu et al. presented data that suggested that there may even be a survival benefit for incomplete resections compared to medical treatment only [45]. Unfortunately, no specifics were given, why the individual cases were deemed inoperable and what was ultimately done. A different approach to challenging cases may be ex-vivo liver resection and autologous transplantation, which has been successfully performed in over 50 cases in China by the group around Wang [46]. Whether this is a feasible approach for inoperable patients we cannot comment on since we have not encountered a case requiring ex-vivo resection yet. In case of unresectable AE lesions that are limited to the liver we wouold rather pursue heterologous liver transplantation, when medical treatment fails.

However, the optimal timing for liver transplantation in AE is hard to define. We recently listed two patients for liver transplantation due to inoperable but symptomatic disease that had been treated with BMZ for years. Unfortunately, both died on the waiting list without receiving an organ offer in time. Given the infectious nature of the disease and the high risk of metastatic disease progression on immunosuppression, clinicians are reluctant to list these patients for transplantation early in the course of disease. Bresson-Hadni et al. reported that the number of patients that underwent LT for AE is decreasing due to the better understanding of medical, surgical and interventional treatment [47].

## 4. Materials and Methods

### 4.1. Data Acquisition

The Department of General, Visceral and Transplantation Surgery of the University of Tuebingen performs around 200 liver resections (80% major) per year in addition to around 50 liver transplantations.

We retrospectively screened our hospital information system for all patients who were treated for Echinococcus disease at our center using the International Classification of Disease Code B67.- Echinococcosis. Here we were able to identify 123 patients. Of these 33 underwent liver resection for Alveolar Echinococcosis at the department of General, Visceral and Transplantation Surgery of the University Hospital of Tuebingen, Germany. All adult patients (age ≥ 16 years) between January 2004 and July 2021 were included in the final analysis. The study was approved by the local ethics committee under the reference number 478/2021/BO2. Individual patient consent was waived by the ethics committee due to the retrospective character of the study.

### 4.2. Clinical Definitions 

The extend of liver resection was classified and described according to the Brisbane terminology of 2000 [16]. Major liver resections are defined as the resection of at least three Coinaud’s liver segments. Atypical resections are liver resections that do not follow the anatomic liver segments.

### 4.3. Histopathology

All liver specimens were examined after hematoxylin-eosin (HE) staining. Additionally, one Periodic Acid Schiff-stain was done on an intrahepatic parasitic lesions for the detection of cuticula. The lymph nodes were routinely stained with HE only. Lymph node infiltration was defined as the detection of cuticula or the parasite itself on the HE stain of within lymphatic tissue. 

### 4.4. Medical Treatment

The gold standard for medical treatment is continuous intake of a benzimidazole. Currently two drugs are used off-label for the treatment of AE. The preferred drug is Albendazole (ABZ) which has a higher bioavailability than it’s older relative Mebendazole. Standard dosing in our cohort was ABZ 10–15 mg/kg/day split in two doses up to a maximum of 800 mg daily. Standard Mebendazole dose is 40–50 mg/kg/day, but this was not prescribed in our cohort. 

### 4.5. Statistics

Statistical analysis was carried out using IBM SPSS Statistics for Windows, Version 27.0 (IBM Corp., Armonk, NY, USA).

## 5. Conclusions

Major liver resection for AE is a challenge that needs to be put in the hands of experienced hepatobiliary and transplant surgeons. Hilar spread can be present even with mainly peripheral lesions and can easily be missed by imaging. Even patients on medical treatment should be periodically revisited with a surgeon to reassess downstaging or the need for salvage resection in case of progression or medical toxicity.

## Figures and Tables

**Table 1:** Patient Characteristics.

Gender male (Percentage)	16 (49%)
Median age in years (range)	54 (19–82)
Body mass index (BMI) in kg/m^2^ (±SD; range)	22.9 (±3.8; 15.8–34.6)
American Soicety of Anesthesia (ASA)-Score ASA 1 ASA 2 ASA 3	2 (6%) 29 (88%) 2 (6%)
Presumed Diagnosis Alveolar Echinococcis Cystic Echinococcosis Intrahepatic Cholangiocarcinoma Colorectal Liver Metastasis	21 (64%) 6 (18%) 3 (9%) 3 (9%)
PNM Stages P1N0M0 P2N0M0 P2N1M0 P3N0M0 P3N1M0 P4N1M0 P4N1M1 (pulm)	5 (15%) 4 (15%) 3 (9%) 2 (6%) 11 (33%) 7 (21%) 1 (3%)
Preoperative Benzimidazole-Treatment Cyclic dosing (28 days) Continuous dosing Median duration in months (±SD; range)	22 (67%) 10 (33%) 12 (36%) 1 (0–146)
Preoperative Benzimidiazole-Toxicity	8 (24%)

Table 1 shows the overall patient characteristics of the study cohort as well as the presumed diagnosis under which the patients were operated upon. Additionally, the postoperatively defined PNM stages and preoperative benzimidazole treatment and toxicity are shown. *Standard Deviation (SD)*.

**Table 2:** Operative Details.

Procedures Atypical Resection Bisegmentectomy Right Hepatectomy Right Trisectionectomy (incl. one Two-stage hepatectomy) Left Hepatectomy Left Trisectionectomy	5 (15%) 4 (12%) 8 (24%) 12 (36%) 3 (9%) 1 (3%)
Additional procedures Lymphadenectomy - Node picking - Radical systematic Portal vein reconstruction Hepatic artery reconstruction Inferior vena cava (IVC) resection - IVC replacement with prosthesis Diaphracmatic resection Extrahepatic bile duct resection >1 Hepatico-Jejunostomy Nephrectomy Lung wedge resection Gastic wedge resection Segmental colectomy Right colectomy Total vascular exclusion Diaphragmatic Argon Plasma Coagulation of parasitic lesion	21 (61%) 6 (18%) 15 (46%) 4 (12%) 2 (6%) 5 (15%) 1 (3%) 4 (12%) 11 (33%) 1 (3%) 1 (3%) 1 (3%) 1 (3%) 1 (3%) 1 (3%) 2 (6%) 1 (3%)
Median operating time in minutes (range) Median length of stay in days (range)	283 (50–530) 11 (3–34)
Pathological Results R0 R1 - R1 (resection margins) - R1 (liver hilum) R2 Hepatic resection margins in mm mean (±SD; median; range)	17 (52%) 14 (42%) 8 (24%) 9 (27%) 2 (6%) 3.5 (±5.4; 1; 0–20)

Table 2 shows the intraoperative extent of surgical procedures, operative time, postoperative length of stay, and resection status.

## Data Availability

The data presented in this study are available on request from the corresponding author. The data are not publicly available du the pseudonymized character of the data.

## Abbreviations

AE	Alveolar Echinococcosis
ABZ	Albendazole
ASA	American Society of Anesthesia
BMZ	Benzimidazole
CE	Cystic Echinococcosis
CRC	Colorectal Carcinoma
CRLM	Colorectal Liver Metastasis
EMA	European Medicines Agency
FDA	Food and Drug Administration
HE	Hematoxylin-Eosin
iCCA	Intrahepatic Cholangiocarcinoma
IVC	Inferior Vena Cava
PHLF	Posthepatectomy Liver Failure
SD	Standard Deviation
